# Propidium Monoazide Combined With RT-qPCR Detects Infectivity of Porcine Epidemic Diarrhea Virus

**DOI:** 10.3389/fvets.2022.931392

**Published:** 2022-07-15

**Authors:** Gong Liang, Yunzhi Long, Qianqian Li, Liu Yang, Ying Huang, Daobing Yu, Wenbo Song, Mingguang Zhou, Gaoyuan Xu, Chao Huang, Xibiao Tang

**Affiliations:** Diagnostic Center Department, Wuhan Keqian Biology Co., Ltd, Wuhan, China

**Keywords:** RT-qPCR, PEDV, PMA, infectivity, examine

## Abstract

Reverse transcription-quantitative polymerase chain reaction (RT-qPCR) allows sensitive detection of viral particles and viruses in epidemic samples but it cannot discriminate noninfectious viruses from infectious ones. Propidium monoazide (PMA) coupled with quantitative polymerase chain reaction (qPCR) was assessed to detect infectious viruses. Currently, there is no established test method to detect the infection of the porcine epidemic diarrhea virus (PEDV). In this study, propidium monoazide coupled with qPCR detects infectivity of PEDV. We optimized the method from the selection of primers, the working concentration of PMA, and the inactivation method using heat or ultraviolet (UV). The viruses which were treated with PMA before qPCR were inactivated using heat or UV. However, the addition of PMA alone did not affect the detection of live viruses, which indicates that a viral capsid break may be essential for PMA to bind to the genome. A repetition of the method on naked PEDV RNA suggests that it can be used to detect potentially infectious PEDV. The results indicated that an optimal plan of PMA could be extremely useful for evaluating infectious and noninfectious viruses.

## Introduction

Porcine epidemic diarrhea virus, a single-stranded RNA virus, is one of the significant pathogens causing porcine epidemic diarrhea, with high mortality in neonatal pigs (1). There were PEDV outbreaks in a few pig-producing provinces in China at the end of 2010 (2). Currently, PEDV infection is widespread in North America, Europe, and Asia. The outbreak of PEDV causes serious economic losses all over the world (3).

The detection methods for PEDV include immunological tests, molecular biological detection, and clinical virus isolation. However, none of the above methods can distinguish between infectious and noninfectious viruses. PMA is a dye with an identification function that can bind to DNA molecules. The dye can enter the small grooves of DNA molecules such as damaged bacteria or viruses. The decomposition of PMA produces substances that covalently cross-link with DNA molecules, thereby inhibiting the PCR amplification of DNA in damaged microorganisms. It has been shown that PMA can penetrate the membrane of compromised cells, leading to a covalent bond formation with the DNA strands, which inhibits subsequent PCR amplification (4, 5). However, PMA cannot penetrate biologically active cells (6). This method has been successfully used for microbiological monitoring of pathogens in a number of previous studies (7–9). PMA was proven to distinguish between infectious and noninfectious poliovirus (10). However, there have been no reported studies of PMA being used in PEDV virus detection to distinguish between infectious and noninfectious viruses.

Given the existing problems in PEDV virus detection, particularly with scant knowledge of the combined application of PMA to detect live bacteria and RNA viruses, this study attempted to use a method based on PMA qPCR technology for the detection of infectious PEDV.

## Methods

### Cell Culture and Virus Amplification

PEDV was extracted from clinical specimens in our laboratory. The clinical specimen was ground and centrifuged. The supernatant was collected, filtered, added to Vero cells, cultured in a 37°C, 5% CO_2_ incubator, blindly passed, and observed for cytopathic effect (CPE). After stable passage for four generations, the cell culture was frozen and thawed three times and centrifuged at 1,700 g at 4°C for 15 min. The supernatant was collected as the PEDV seed and stored at 4°C. The PEDV strain was then selected, inoculated into Vero cells, cultured at 37°C, 5% CO_2_, and cultivated in the box for 3 days. It was observed for cell CPE, after which the virus liquid was filtered, collected, and diluted 10-fold.

### Inactivation and CPE Assay

The virus was inactivated in a water bath at 100°C for 30 min. For UV inactivation, the virus was subject to ultraviolet irradiation for 4 h. The infectivity of PEDV after inactivation by heat or UV was determined using CPE assays. The virus was inactivated, planted into cells, and cultured in an incubator for 3 days. Then, the cells were examined under a microscope.

### Viral Infectivity Assays

To determine the effectiveness of different inactivation methods, the PMA working solution was added to live PEDV, the heat-inactivated PEDV, and the nucleic acids of PEDV or the UV-inactivated PEDV. To determine the effectiveness of different concentrations of PMA, the final concentrations of PMA were set as 10, 20, 50, and 100 μM. To assess the effectiveness of different primers, *PEDV-S* and *N* primers were used. In order to test the specificity of the primers and the inactivation methods, the recombinant plasmid pUC19-PEDV-N standard was prepared, and the amplification curve was established (11). The PEDV and control viruses such as swine fever virus, porcine pseudorabies virus, and porcine circovirus were selected as samples. Water was used as the control group.

Samples of different treatments underwent PMA assays. PMA was mixed with samples and treated with a protective light for 15 min and then with the blue light of 100 W for 20 min, at the same time, and shaken one time at intervals of 5 min.

### Virus RNA Extraction and RT-qPCR

The viral genome extraction kit was used for DNA extraction and PCR verification. Extracted RNA was thawed before the RT-qPCR assay. Primers for PEDV are presented in Table 1. The CFX96TM real-time PCR instrument was used for detection.

**Table 1:** Primer.

**Amplified genes**	**Primer sequences (5' → 3')**	**Annealing temperature/**°**C**	**Product length/bp**
***PEDV-N***	F: AGCAACAGCAGAAGCCTAAGCA	56	232
	R: GCATAGCCTGACGCATCAACAC		
***PEDV-S***	F: CCTGCGTTCGGTAGTGGTGTTAA	56	139
	R:TATACTTGGTACACACACATCCAGAGTCA		

### Statistical Analysis

Statistical comparisons were performed by the one-way analysis of variance (ANOVA). Each experiment was repeated at least three times and fluorescence signals were collected. The criteria for determining the detection of viruses were as follows:

(1) When the sample Ct value is ≤ 35, the sample was considered to be positive.
(2) If the Ct value ranges between 35 and 40, and if the logarithmic amplification curve is curved, then the sample was judged to be suspicious positive. Alternatively, the sample was judged to be negative and the test was repeated. If the Ct value of the retested sample is ≤ 35, the suspicious sample was judged as positive.
(3) If the sample has no Ct value or Ct value > 40, the sample is judged to be negative.

## Results

### CPE Assay

**Figure 1:**
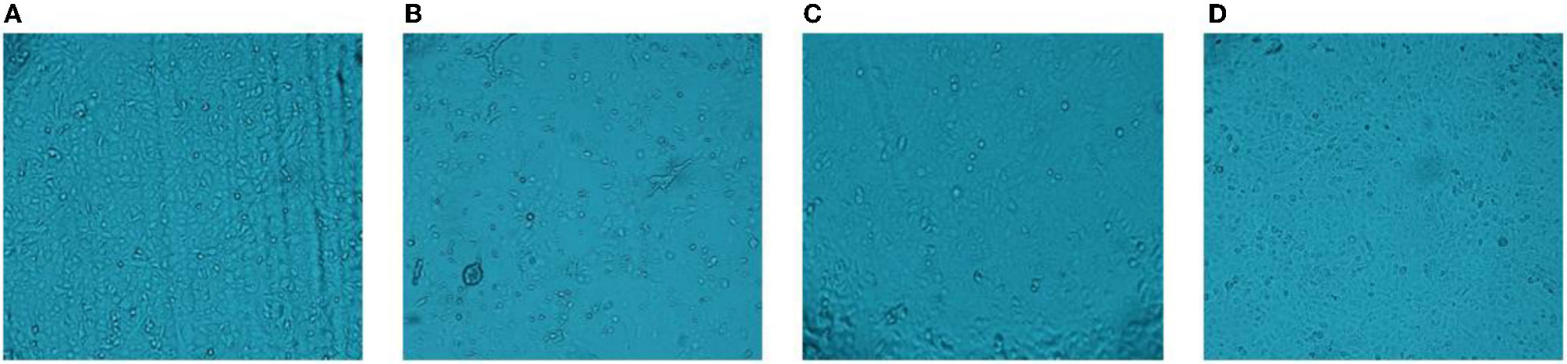
CPE assay. **(A)** blank group; **(B)** cytopathic effect; **(C)** heat treatment; **(D)** UV treatment.

The CPE were not observed in this blank control group (Figure 1A). Figure 1B shows that the virus can cause cell lesions compared to the blank control group when the virus is untreated. When the PEDV was inactivated by heat, it did not display CPE (Figure 1C). The UV-inactivated group too did not have CPE (Figure 1D). It shows that PEDV can be effectively inactivated by heat or UV treatments.

### The Influence of PMA QPCR on the Detection of PEDV

**Figure 2:**
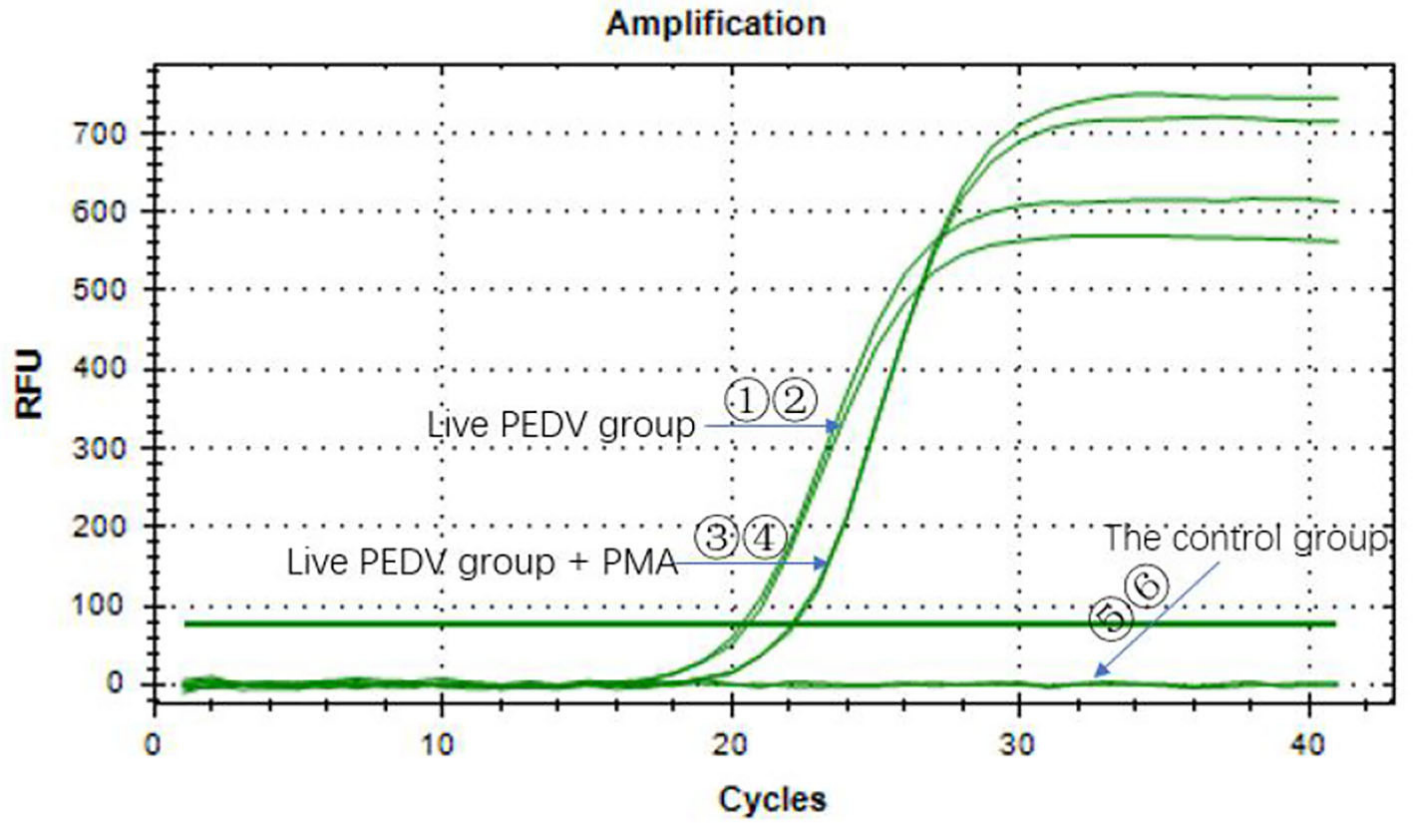
Effect of PMA on the qPCR detection of porcine pseudorabies virus.

The Ct values of the two groups with the PEDV live virus (①②) were 20.56 and 20.36. The Ct values of the two groups with PMA added to the PEDV live virus (③④) were both 22.07, which was <35, indicating that the sample was positive (Figure 2). The control group did not have a Ct value, and the sample was negative. It indicated that the PMA had a minor effect on the PEDV virus detection results. Overall, PMA could be used for PEDV virus detection.

### PMA QPCR on Naked Viral RNA

As shown in Table 2, PMA can effectively inhibit the amplification of naked viral nucleic acid (*P* < 0.05).

**Table 2:** The effectiveness of PMA.

**Group**	**Ct**
The control group	35.43 ± 0.51^a^
PEDV	25.72 ± 0.34^b^
PEDV+PMA	35.52 ± 0.42^a^

*Different letters indicate significant differences (a–c) (P <0.05)*.

### PMA RT-qPCR on Infectious and UV-Inactivated Viruses

These results indicate that the PMA treatment to differentiate between infectious and UV-inactivated viruses had no effect (The result was marginal).

### Optimization of Primers and the Concentration of PMA

To determine the effectiveness of different primers, the primers of *PEDV-S* and *N* were tested. When using the *PEDV-S* primers in the final concentration of PMA, the sample Ct value was ≤ 35 (Table 3). Therefore, the primer of *PEDV-S* could not be used to determine whether the PEDV virus was infectious. In the *PEDV-N* primers, the live and heat-inactivated *PEDV* were detected by the PMA qPCR, and the Ct values were <35 when the final concentration of PMA was 10 and 20 μM (Table 3). When the final concentrations of PMA were 50 and 100 μM, the Ct value of the PEDV live virus also was <35, but it was more than 35 in PEDV heat-inactivated virus. When the final concentration of PMA was 50–100 μM, *PEDV-N* primers were used for qPCR amplification to distinguish between PEDV live viruses and PEDV heat-inactivated virus.

**Table 3:** Two primers to determine the effectiveness of different PMA concentrations.

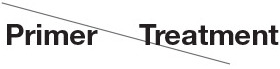	**Virus inactivation**	**10 μM**	**20 μM**	**50 μM**	**100 μM**
*PEDV-S*	No	+	+	+	+
	Yes	+	+	+	+
*PEDV-N*	No	+	+	+	+
	Yes	+	+	-	-

### Specificity of Primers

**Figure 3:**
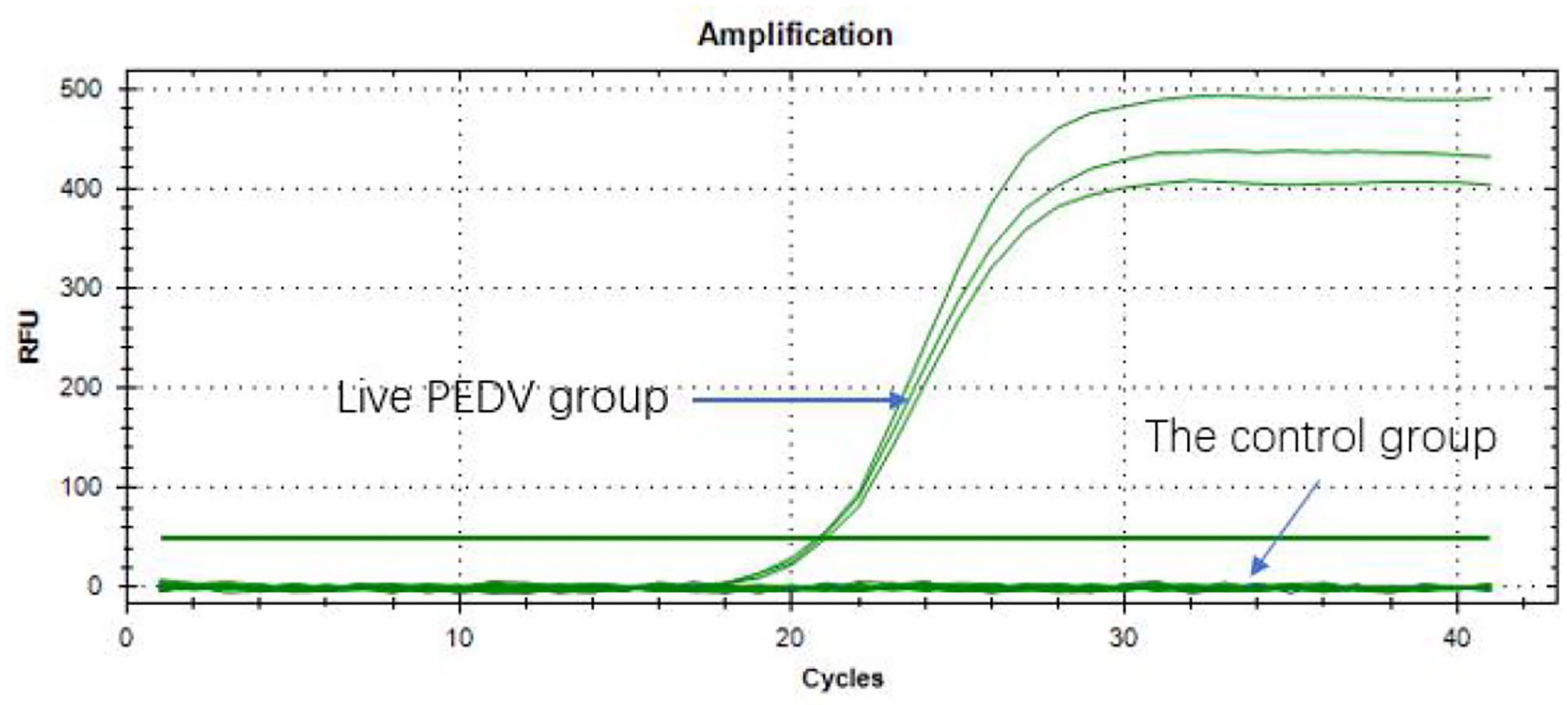
Specificity of primers.

The qPCR amplification was performed in all the PEDV live virus groups. The results had a Ct value of about 20 and <35, indicating that the samples were positive (Figure 3). The control viruses did not have a Ct value, and the results of heat/UV -inactivated samples were all negative, which indicated that the PMA qPCR method, designed for the first time in this study, had obvious specificity and showed accuracy in detecting PEDV.

**Figure 4:**
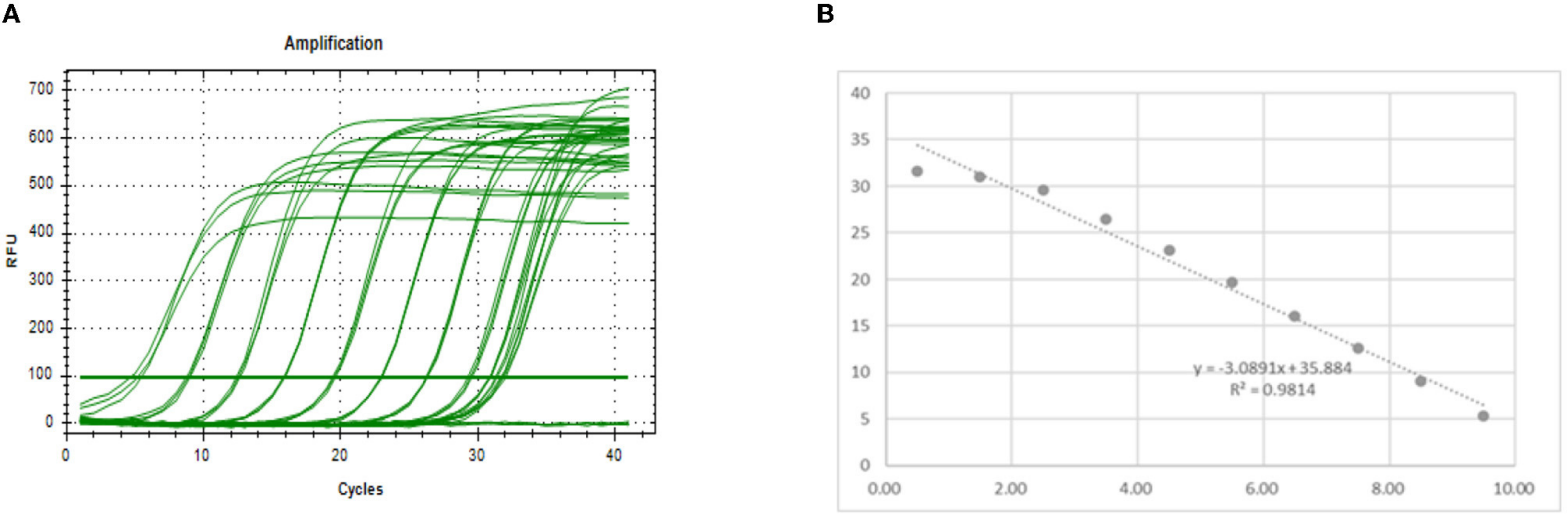
Drawing a standard curve. **(A)** Amplification curve. **(B)** Standard curve.

The preparation method of the standard substance is to insert the PEDV-N gene fragment into PUC19 by using the amplification primer determined in the previous step to form the recombinant plasmid. After the copy number of the recombinant plasmid concentration prepared thus was measured, it was diluted 10-fold and the qPCR amplification was performed. The amplification results are shown in Figure 4A. The regression equation of the standard curve was y = −3.0891x + 39.278. According to the formula, E = 10^−1/*K*^-1 (where E is the amplification efficiency and K is the slope of the standard curve), the amplification efficiency was 110.73%, indicating that the primers provided had sufficient sensitivity (11).

### Effectiveness of Different Virus Dilution Ratios

The virus stock had a Ct value of 28.15 when was used for amplification. It changed to 30.05 when it was diluted 10-fold (Table 4). According to the standard curve regression equation used in Figure 4B (y = −3.0891x + 39.278, where x represents the logarithm of copy number and y is Ct value; R^2^ = 0.9814), when the virus stock was amplified, the Ct value was the same (28.15) and the copy number was 4002.62 copy/μL. When virus stock was diluted 10-fold, the Ct value was 30.05, and the copy number was 971.13 copy/μL. The highest copy number of PEDV detected by PMA qPCR was 971.13 copy/μL. Therefore, in the case of infectious PEDV detected by PMA qPCR (when the virus was diluted 10-fold, and the copy number of the virus was 971.13 copy/μL), the primers designed by the N gene were used for qPCR amplification to distinguish whether PEDV was infectious or not.

**Table 4:** Effectiveness of different virus dilution ratios.

**Dilution**	**Infectivity**	**Non-infectivity**
Control	0	0
1	28.15 ± 0.13^c^	30.42 ± 0.31
10	30.05 ± 0.41^b^	0
100	33.42 ± 0.54^a^	0
1000	0	0

*Different letters indicate significant differences (a–c) (P <0.05)*.

### PMA RT-qPCR on Infectious and Heat-Inactivated Viruses

**Figure 5:**
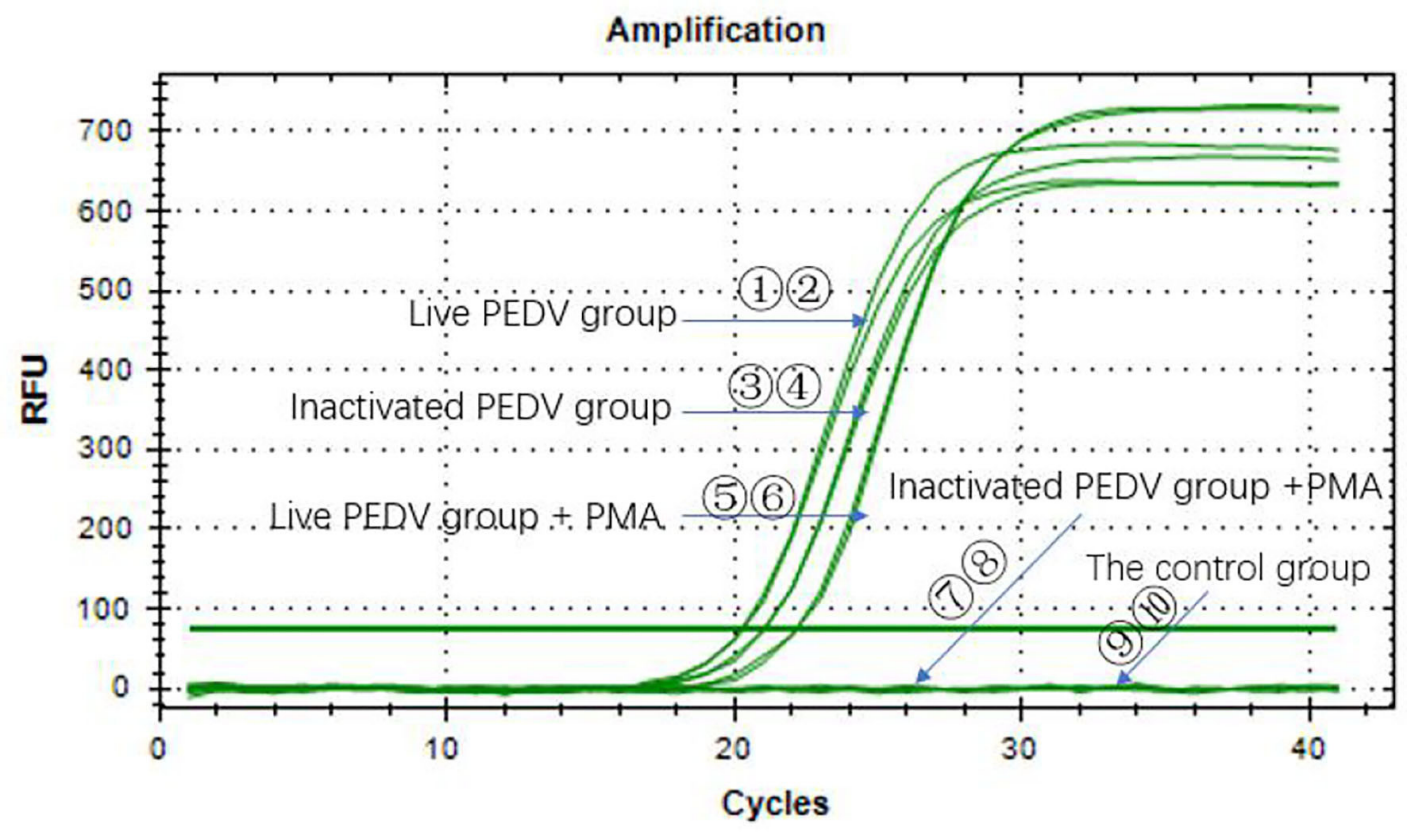
PMA RT-qPCR on infectious and heat-inactivated viruses.

Figure 5 shows that the Ct values of the PEDV live virus in two groups (①②) were 20.30 and 20.77, respectively. The Ct values (⑤⑥) of PEDV live virus +PMA were 22.18 and 22.17, respectively. The Ct values of the PEDV heat-inactivated groups (③③) were 21.06 and 21.05, respectively. The Ct values of the PEDV live virus group, the PEDV live virus +PMA group, and the heat-inactivated PEDV virus group were <35, which indicated that the samples were all positive. The inactivated PEDV virus + PMA group (⑦⑧) and the water control group (⑨⑩) did not have a Ct value, and the samples were negative. The results indicated that the method presented in this study can effectively detect infectious PEDV.

## Discussion

The outbreak of PEDV causes serious economic losses throughout the world. Currently, the detection methods for PEDV include immunological tests, molecular biological tests, and clinical virus isolation. However, immunological tests that cannot detect the infectivity status of the microorganisms are problematic. Therefore, the need to develop a rapid, accurate, and simple method that detects infectious PEDV and conducts real-time monitoring of PEDV is rather urgent.

The nucleic acid detection methods based on PCR, such as PCR, qPCR, RT-LAMP, and RT-RPA, have the advantages of high detection sensitivity, strong specificity, and fast detection speed. A double ultrasensitive nanoparticle DNA probe-based PCR assay for testing and distinguishing diagnosis of PEDV (12) is also available. There also exists a fast differentiation of the two strains of PEDV *via* an amplification assay (13). However, none of the above methods can distinguish between infectious and noninfectious viruses. Additionally, the PEDV test used in the sample is likely to lead to false-positive results because the virus genome can still be detected for a short period of time after the virus dies. PMA in RT-PCR has been used previously to detect infectious enteric viruses (14, 15).

With regard to the concentration of PMA to be used, in the present study, we found that a concentration of 50–100 μM is effective (15, 16). Karim et al. (16) reported that PMA was able to differentiate between infectious and noninfectious murine norovirus (MNV) only when inactivation was done by chlorine. The addition of PMA alone does not affect the detection of live viruses. While UV prevents the virus from replicating, it does not destroy the cell membrane of the virus. PMA, therefore, cannot still enter the virus cell and bind to the viral genome, and there is no way to identify the infectivity of the virus. The varying efficiency of PMA to distinguish noninfectious from infectious viral particles might be owing to the degree of RNA or capsid damage induced by each inactivation method. In our study, PEDV was heat treated with a water bath at 100°C for 30 min and PEDV lost its infectivity. The results also showed that PEDV lost its infectivity when exposed to UV. It has been proven that the PMA binding mechanism is critical to distinguishing between infectious and noninfectious microorganisms (17–21). PMA's comparable effectiveness in detecting infectious PEDV when treated by heat or UV was examined in this study.

The PMA coupled with RT-qPCR developed in this study specifically targets PEDV, providing a useful method for clinical diagnostic laboratories. The frequent spread of African Swine Fever (ASF) has received a high level of attention due to the threat posed to pig farming worldwide (22). There is a need, therefore, to establish an optimal way of determining the infectivity of ASF.

## Conclusion

PMA combined with RT-qPCR could be very effective for determining infectious and noninfectious viruses.

## Data Availability Statement

The datasets presented in this article are not readily available because requests to access the datasets should be directed to GL, 2731327322@qq.com.

## Author Contributions

CH and XT conceived the project, analyzed the data, and revised the manuscript. GL designed and performed the experiments, analyzed the data, and wrote the manuscript. YL, LY, WS, YH, DY, MZ, and GX wrote sections of the manuscript. All authors contributed to the article and approved the submitted version.

## Funding

This study received funding from Wuhan Keqian Biology Co., Ltd. The funder was not involved in the study design, collection, analysis, interpretation of data, the writing of this article, or the decision to submit it for publication.

## Conflict of Interest

All authors were employed by Wuhan Keqian Biology Co., Ltd. The authors declare that the research was conducted in the absence of any commercial or financial relationships that could be construed as a potential conflict of interest.

## Publisher's Note

All claims expressed in this article are solely those of the authors and do not necessarily represent those of their affiliated organizations, or those of the publisher, the editors and the reviewers. Any product that may be evaluated in this article, or claim that may be made by its manufacturer, is not guaranteed or endorsed by the publisher.
